# Design and Evaluation of RNA Aptamer–Mediated Delivery of C/EBP*β* siRNA for Oncological Therapy

**DOI:** 10.1155/jna/1461574

**Published:** 2025-03-03

**Authors:** D. Vasconcelos, M. H. Sodergren, V. Reebye, J. Vasara, M. S. Song, K. Holm, S. E. Khorsandi, J. Rossi, N. Habib, K. W. Huang

**Affiliations:** ^1^Department of Surgery and Cancer, Imperial College London, London, UK; ^2^Apterna Ltd., London, UK; ^3^Department of Molecular and Cellular Biology, Beckman Research Institute of City of Hope, Duarte, California, USA; ^4^Department of Surgery and Hepatitis Research Center, National Taiwan University Hospital, Taipei, Taiwan (ROC)

## Abstract

The CCAAT/enhancer-binding protein beta (CEBPB or C/EBP*β*) is a transcription factor that plays a critical role in cellular differentiation, metabolism, and immune response. Emerging evidence has highlighted its complex involvement in both solid and hematological cancers, such as hepatocellular carcinoma (HCC) and pancreatic ductal adenocarcinoma (PDAC), where it can act as an oncogene or a tumor suppressor, depending on the context. In this study, we describe the design and evaluation of a conjugate formed by a small interfering RNA (siRNA) for CEBPB and a transferrin receptor targeting aptamer (TfR-siCEBPB). The designed conjugate is active in human and mouse cells, by transfection and by passive uptake, demonstrating target engagement with strong downregulation of CEBPB mRNA transcript. In murine models of metastatic PDAC and cirrhotic HCC, treatment with TfR-siCEBPB was associated with reduction in tumor burden and improvement in liver function biomarkers. This novel aptamer conjugate allows delivery of targeted oligonucleotide therapy and is a promising therapeutic tool to take forward to human trials.

## 1. Introduction

CCAAT-enhancer-binding proteins (or C/EBPs) are a family of transcription factors that interact with the CCAAT box motif gene promoter and thereby modulate gene expression [1]. These transcription factors control many biological processes, including cell growth, differentiation, metabolism, and death [1]. It was previously shown, in preclinical and clinical studies, that upregulation of C/EBP*α* (CEBPA), using a small activating RNA (saRNA), is an effective novel immunotherapy by its ability to target the differentiation of myeloid-derived suppressor cells (MDSCs) to produce antitumor effect [2–5]. Downregulation of C/EBP*β* (CEBPB) is an alternative way to upregulate CEBPA [6]. In contrast to CEBPA, an increase in CEBPB expression has been associated with worse oncological outcomes in several tumor types including prostate [7], breast [8], and colon [9].

Hepatocellular carcinoma (HCC) and pancreatic ductal adenocarcinoma (PDAC) are two types of cancer with high mortality rates and limited treatment options [10–12], with a 5-year survival of 18% and less than 5%, respectively. Despite improvement in systemic therapies, with the introduction of sorafenib [13] for HCC and FOLFIRINOX for PDAC [14], and more recently immune checkpoint inhibitors (ICIs) [15, 16], the median overall survival for these patients still remains dismal [12, 14]. The combination of targeted therapies with immunotherapy in the form of ICIs such as programmed cell death 1 (PD-1) has led to dramatic improvements in clinical outcomes for many cancers, but the results of monotherapy, namely, in PDAC, have been rather disappointing [17].

The role of CEBPB and its isoforms in cancer is complex and context dependent. In hepatic carcinoma, specifically HCC, CEBPB has been shown to either promote or inhibit hepatic carcinogenesis. Depending on the relative expression levels of its isoforms (LAP1, LAP2, and LIP) [18], it affects regulation of genes involved in hepatocyte proliferation, apoptosis, and differentiation [4, 19]. Higher expression of CEBPB has also been observed in PDAC tissues, and this overexpression has also been associated with a poorer prognosis [20]. PDAC is characterized by a dense stromal reaction and a highly desmoplastic environment, where CEBPB has been found to contribute to the fibrotic response and to modulate the tumor microenvironment [20]. Moreover, CEBPB has been linked to the regulation of genes involved in the epithelial–mesenchymal transition (EMT), a process that is critical for cancer cell invasion and metastasis [21].

Targeting the human transferrin receptor 1 (TfR) to deliver therapeutic oligonucleotides is a promising strategy for treating liver and pancreatic cancers, due to the overexpression of TfR on the surface of cancer cells [22], including HCC [23] and metastatic PDAC [24] cells. This overexpression facilitates receptor-mediated endocytosis, which can be exploited to deliver therapeutic agents directly into various types of cells [25, 26], in particular cancer cells [22]. The TfR protein sequence similarity between human and mouse, as well as rat, is 76%, which has allowed a good translation between in vitro and in vivo activity, in several of our previous studies [27, 28].

In previous work, we demonstrated that conjugates of RNA aptamers with saRNAs, P19-saCEBPA [29] and TfR-saCEBPA [27], have potent antitumor effects in a pancreatic cancer mouse model. Aptamers are short, single-stranded oligonucleotides, which can bind to a specific target molecule, with high affinity and specificity. Aptamers are selected by a process known as systematic evolution of ligands and exponential enrichment (SELEX) [30, 31], which can be driven to identify cancer cell–specific sequences [32]. Previously, we employed a multilevel in silico protocol, including protein–aptamer docking, molecular dynamics simulations, and free energy calculations, which revealed details at the molecular level, such as the aptamer binding to the receptor and the impact of conjugation with a short therapeutic RNA [33].

In this study, we describe the design of a conjugate formed by a small interfering RNA (siRNA) for CEBPB mRNA transcript (siCEBPB) and a TfR RNA aptamer and evaluate its activity in vitro and in vivo, namely, in murine models of primary cirrhotic HCC and PDAC with liver metastasis. Using both primary and secondary liver cancer models enables a more comprehensive understanding of the TfR-siCEBPB conjugate therapeutic potential.

## 2. Materials and Methods

### 2.1. Design of siRNA Oligonucleotides and TfR Aptamer Conjugates

The methodologies for identification and screening of siRNAs for targeted gene knockdown have been previously described [34–36]. Selection and optimization of the TfR aptamer were previously described by our group [27]. Conjugates were designed and synthesized with TfR aptamer sequence at the 5⁣′ end and at the 3⁣′ siRNA sense strand. Chemically modified sequences were designed using a similar pattern to enhanced stability chemistry (ESC) [37]. In the applied pattern, all the 2⁣′-OHs are substituted with an alternating 2⁣′-O-methyl (2⁣′-OMe) and 2⁣′-Fluoro (2⁣′-F) pattern, with three repeated nucleotides at Positions 7–9 for passenger and 11–13 for guide strand. The two terminal phosphate bonds are substituted with phosphorothioates (PS), at each 3⁣′ end of the passenger and guide strands and at 5⁣′ end of guide [37]. Human (h), mouse (m), mismatch converted human/murine cross-reactive (h/m)siCEBPB, and negative control siFLUC sequences are presented in Table 1.

### 2.2. Transferrin Aptamer Synthesis and Conjugation to siRNA

The TfR-siCEBPB sense and antisense strands were chemically synthesized *in house* by solid phase oligonucleotide synthesis. The 48-residue long aptamer-spacer-sense strand was completely synthesized by phosphoramidite chemistry, as a single-stranded RNA oligonucleotide, in a one pot reaction. The average yield for the full conjugated strands was 65% while for the antisense strands was 90%. HPLC purification of compounds was achieved by IP-RP HPLC using a DNAPac RP column, 4 *μ*m, 150 mm × 10 mm, kept at 60°C, using buffer A, 100 mM triethylammonium (TEAA) in H_2_O and buffer B, 100 mM TEAA in 50% acetonitrile, under a linear gradient 20%–70%, at 1 mL·min^−1^. Identity was confirmed by ESI-MS and purity was > 95% for all compounds. Aptamer sense and complementary antisense strands were then mixed at the same molar amounts, annealed and refolded in Dulbecco's DPBS + 0.9 mM Ca^2+^ + 0.5 mM Mg^2+^ buffer, heated to 95°C for 5 min, incubated at 37°C for 30 min, and finally slowly cooled to room temperature. Annealing of both strands was confirmed by PAGE using precasted 20% acrylamide Novex gels (Invitrogen, EC6315).

### 2.3. In Vitro Activity of siCEBPB

To test the activity of (h/m)siCEBPB, three cell lines were used, (i) PANC-1 (ATCC, CRL-1469) for human transcript activity and cross-reactivity, (ii) primary mouse hepatocytes (PMHs) (Thermo Fisher, MSCP10) for mouse cross-reactivity and dose response, and (iii) mouse liver epithelial BNL 1ME cells (ATCC, TIB-75), for testing ESC stabilized sequence activity. PANC-1 cells, a human cell line established from a pancreatic carcinoma of ductal origin, were cultured in antibiotic free DMEM + 2 mM glutamine + 10% fetal bovine serum (FBS) (Thermo Fisher) and detached using Accutase detachment reagent (Invitrogen, 00455556). Cells were seeded at 5 × 10^4^ cells per well in a 24-well plate using 500 *μ*L of media and incubated at 37°C, 5% CO_2_. Twenty-four hours post cell seeding, siRNAs were added to cells after complexation with Lipofectamine 2000 (L2K) (Thermo Fisher, 11668019), prepared according to manufacturer's protocol, or as free TfR-siRNA, from 25 *μ*M working solutions, incubated for 48 h. Cells were then lysed and harvested for further analysis.

To test cross-reactivity to mouse mRNA transcript, siRNA sequences were tested in PMHs. Cryopreserved PMHs were thawed and seeded, according to the supplier's protocol, in collagen-coated, 24-well plates at 5 × 10^5^ cells/well. Twenty-four hours post seeding, TfR-siRNA conjugates were added to cells, in the absence of a transfection agent, at 1, 10, 100, and 1000 nM, from freshly prepared 25 *μ*M working solutions and incubated for 48 h, after which cells were lysed and harvested for further analysis.

Activity in mouse cells of the chemically modified sequences, designed using a similar pattern to ESC (“ESC similar”) [37], was tested using BNL 1ME cells, a mouse liver epithelial cell line. Cells were maintained in complete DMEM, seeded at 2.5 × 10^4^ cells per well in a 24-well plate using 500 *μ*L of media, and incubated at 37°C, 5% CO_2_. Twenty-four hours post seeding, siRNAs complexed with L2K, prepared according to manufacturer's protocol, were added to cells at 1, 5, 10, and 20 nM and incubated for 48 h, after which cells were lysed and harvested for further analysis.

For CEBPB mRNA knockdown analysis, total cell RNA was extracted for reverse transcription using RNeasy kit (Qiagen). Target cDNA amplification and real-time PCR were performed using SYBR Green PCR Master Mix (Applied Biosystems) and target-specific QuantiTect Primer Assays (Qiagen) (CEBPB: Hs_CEBPB_1_SG, QT00237580; Mm_Cebpb_1_SG, QT00320313; B2M: Hs_B2M_1_SG, QT00088935; Mm_B2m_2_SG, QT01149547; HPRT: Hs_HPRT1_1, QT00059066; Mm_Hprt_1_SG, QT00166768).

### 2.4. Oligonucleotide Stability Assay

To test if chemically modified sequences became more resistant to activity from nucleases, samples were incubated with RNAse I or FBS to mimic in vivo conditions. Unmodified and chemically modified siRNA sequences, designed using a similar pattern to ESC, were incubated with 10% FBS (Thermo Fisher Scientific, A3160801) or with 0.5 units of RNAse I (Ambion, AM2294) for 0 min, 5 min, 30 min, and 1 h at 37°C on a thermomixer. (m)siCEBPB, (h/m)siCEBPB, and correspondent “ESC similar” stabilized samples were snap frozen and kept at −80°C until ready for electrophoresis run. TriDye ultralow range DNA ladder (NEB, N0558S), microRNA marker (NEB, N2102S), and samples were loaded into a 20% polyacrylamide Novex TBE gel (Invitrogen, EC6315) and ran at 170 V for 75 min. Afterwards, gels were incubated in 1:10,000 SYBR Gold (Thermo Fisher Scientific, S1149) in 1x TBE for 20 min on a shaker, in the dark. Finally, images were obtained with Li-Cor D-Digit (LicorBio, Germany) at 1x exposure.

### 2.5. Animal Models, Experimental Design, and Sample Collection

Animal studies were performed in compliance with approval from the Institutional Animal Care and Use Committee of College of Medicine, National Taiwan University. Animals were given standard chow fed and kept in a conventional, specific pathogen-free facility, at a maximum of three per cage, with a standard controlled environment temperature (22 ± 3°C), 50 ± 20% humidity, light/dark cycle of 12 h per day, and 15–20 fresh air changes per hour.

### 2.6. Pancreatic Cancer Liver–Metastatic Mouse Model (PDAC)

Development of intrahepatic pancreatic cancer liver–metastatic mouse model of PDAC was previously described in detail [27]. Briefly, to establish the model, an intrahepatic tumor implantation was performed, by injecting 30 mL of a monocellular suspension (in phosphate buffer saline (PBS)) containing 1 × 10^6^ PANC-1-Luc2 cells (ATCC, CRL-1469-LUC2), into a region in the middle lobe of the livers of 6-week-old NOD/SCID male mice (BioLasco). Tumors were allowed to grow for 2 weeks after inoculation; then, mice were randomly divided into five groups of five animals and injected subcutaneous (s.c.) with 100 *μ*L of PBS, TfR-siFLUC, TfR-(m)siCEBPB, or TfR-(h/m)siCEBPB, all formulated in PBS. siRNAs were all chemically stabilized with “ESC similar” pattern. Administered dose was 15 mg/kg, with injections at Days 1, 3, 5, 8, 11, 14, and 17. Tumor growth was monitored by evaluating bioluminescence as photon counts, using an IVIS 200 in vivo imaging platform (Caliper Life Sciences). Prior to imaging, mice were anesthetized using isoflurane and an intraperitoneal (i.p.) solution of 150 mg/kg D-Luciferin Firefly, potassium salt (Biosynth, L-8220) was injected. Bioluminescent signals were analyzed using Living Image Software (Caliper Life Sciences). Animals were culled at Day 21 after first injection; livers were excised; and tumors were dissected out, weighted, and measured with a Vernier caliper for volume determination, using the formula *V* = *a* · *b*^2^/2 (*a* is the largest diameter; *b* is the smallest diameter). Serum and tissue samples were snap frozen for further analysis.

### 2.7. Rat Chronic Liver Disease Model and Primary HCC

HCC was established on a background of chronic liver disease, and tumor burden was assessed as previously described [3]. The diethylnitrosamine (DEN, C_4_H_10_N_2_O) chronically induced rat model reproduces human liver fibrosis and cirrhosis, leading to HCC development [3]. In brief, male Wistar rats were given 100 ppm (vol/vol) of DEN solution in drinking water, over a course of 9 weeks. The concentration of DEN in solution was adjusted in proportion to the body weight of the animals. After 9 weeks of DEN exposure, there was a washout period of 3 weeks, where animals were given normal water—thus providing sufficient time required for spontaneous liver tumors to be developed [38]. At the end of 12 weeks, animals were randomized into two groups of eight, according to their body weight. Following a previously tested protocol [27], animals were treated via s.c. injection with PBS or 15 mg/kg of (h/m)TfR-siCEBPB (ESC), once weekly for 3 weeks. Tumor burden, in terms of volume and weight, was determined as described above and previously [3]. Survival rate was monitored and serum biomarkers for liver function were analyzed from serum collected from alive animals at time of culling. Measured biomarkers and reference values are as follows: low-density lipoprotein (LDL, 20–50 mg/dL), high-density lipoprotein (HDL, 21–54 mg/dL), aspartate aminotransferase (AST, 50–200 U/L), alanine aminotransferase (ALT, 25–75 U/L), bilirubin (0.2–0.7 mg/dL), albumin (ALB, 3.1–4.6 g/dL), and glucose (GLU, 50–120 mg/dL) [39].

### 2.8. Tissue RNA Extraction and Real-Time PCR

Biopsied tumor and background liver tissue, from five animals of each group, were dissected into approximately 25-mg fragments on dry ice and preserved in RNAlater (Thermo Fisher Scientific) at −80°C. The day before RNA extraction, samples were left to thaw at 4°C. Each sample was dried with a Kimwipe and two punches (~5 mg) were taken using a 1.5-mm tissue plunger. The tissue punches were immediately transferred into a SureLock tube containing 350 *μ*L of Qiazol and metal beads. The tissue was then disaggregated with a TissueLyser II unit (2 min, 20 Hz). Upon complete tissue disruption, 150 *μ*L of 1-bromo-3-chloropropane was added, and then, samples were vortexed thoroughly for 20–30 s and spun in a prechilled centrifuge at 4°C, 13200 rpm for 15 min. The aqueous upper layer was then transferred into an RNAeasy mini column for RNA extraction following Qiagen protocol. Total RNA concentration was quantified using a QIAxpert spectrophotometer (Qiagen) and 500 ng reverse transcribed using the Quantitect Reverse Transcription Kit (Qiagen). Relative expression levels were determined by qPCR on an ABI 7900HT thermal cycler (Applied Biosystems), using Quantifast SYBR Green Master Mix (Qiagen) and target-specific QuantiTect Primer Assays (Qiagen) (CEBPB: Rn_Cebpb_1_SG, QT00366478; CEBPA: Rn_Cebpa_1_SG, QT00395010; B2M: Rn_B2m_1_SG, QT00176295).

### 2.9. Statistics and Analysis

Data was analyzed with GraphPad Prism software, version 9.0.1, using Student's *t*-test, ANOVA, or unpaired *t*-test with Welch's correction to assess statistical significance (⁣^∗^*p* < 0.05, ⁣^∗∗^*p* < 0.01). IC_50_ values were determined from dose response curves, with nonlinear regression analysis using “dose-response-inhibition” function of GraphPad Prism.

## 3. Results

### 3.1. TfR-siCEBPB Activity in Human and Mouse Cell Lines

**Figure 1:**
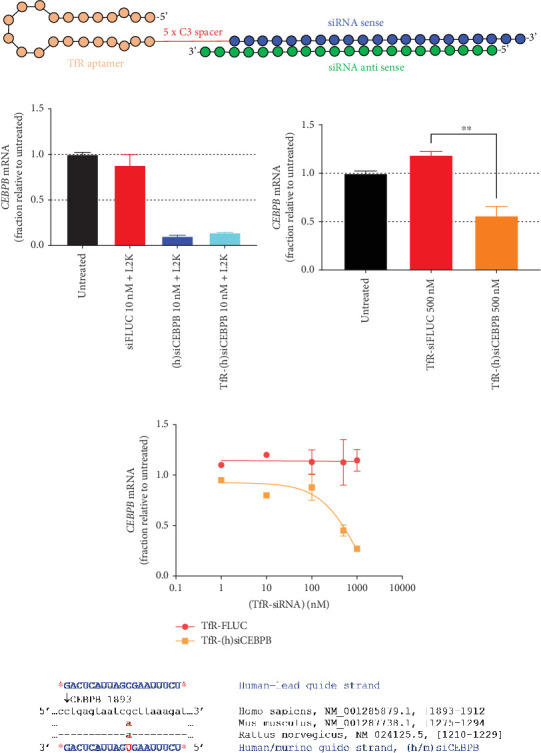
In vitro activity for TfR-(h)siCEBPB in PANC-1 and primary mouse hepatocyte cells. (a) Schematic representation of TfR-siRNA conjugates. (b) PANC-1 cells were transfected 24 h after seeding with siFLUC negative control, human-specific (h)siCEBPB or TfR-(h)siCEBPB at 10 nM. (c) PANC-1 cells were directly treated with 500 nM of TfR-siFLUC or TfR-(h)siCEBPB, without a transfecting agent. (d) Cryopreserved primary mouse hepatocytes (PMHs/MSCP10) were treated with varying concentration of TfR-siFLUC or TfR-(h)siCEBPB, in the absence of transfecting agent. CEBPB mRNA transcript level was measured by qPCR. (e) (h)siCEBPB targeting region shows single mismatch between human and murine. Mismatches to human targeting site are shown in red. Guide strand of human/murine (h/m)siCEBPB converted mismatch. Statistics: asterisks denote significance as follows: ⁣^∗∗^*p* < 0.01; unpaired *t*-test with Welch's correction; *n* = 2, mean ± SD; *n* = 1 for PMH.

Using the same chemical scaffold for all the TfR-siRNA conjugates (Figure 1(a)), a series of experiments were performed to test its activity in vitro. Cellular delivery and CEBPB mRNA silencing activity for (h)siCEBPPB were tested by L2K-mediated transfection and by passive uptake using TfR-(h)siCEBPB (Figure 1). PANC-1 cells, L2K transfected with 10 nM of (h)siCEBPPB, showed a significant reduction of ~90% CEBPB mRNA expression, relative to control. To confirm that the same (h)siCEBPPB sequence would retain its activity when conjugated to the TfR aptamer, PANC-1 cells were L2K transfected with 10 nM of TfR-(h)siCEBPB, which in its turn resulted in ~85% knockdown of the target mRNA (Figure 1(b)). In addition, TfR-(h)siCEBPB was added directly to PANC-1 cells at 500 nM to confirm activity by passive uptake, which resulted in a reduction of ~50% of CEBPB mRNA, relative to untreated (Figure 1(c)). No visible signs of cytotoxicity were observed for any of the conditions.

In order to confirm the activity of (h)siCEBPB against the mouse transcript, PMHs were treated directly with TfR-(h)siCEBPB, which resulted in a significant reduction of ~70% target mRNA at 1 *μ*M, relative to control, and an IC_50_ of ~0.7 *μ*M (Figure 1(d)), thus confirming the sequence silencing activity, both for human and mouse transcripts.

To design a mismatch converted surrogate of the human sequence (NM_001285879.1) to cross-react with murine mRNA, a pairwise sequence alignment was done using NCBI nucleotide BLAST, for the human (h)siCEBPB sequence against the mouse (NM_001287738.1) and rat (NM_024125.5) CEBPB transcripts. A single mismatch was found at the targeted region, which suggests a highly conserved sequence (Figure 1(e)) (Table 1).

### 3.2. Sequence Cross-Reactivity and Stabilization Through Chemical Modification of siCEBPB

Based on the successfully tested (h)siCEBPB sequence, we proceeded to optimize the sequence, namely, by applying a mismatch conversion strategy, aiming to enhance activity and stability in vivo. To find out if activity in mouse cells could be improved, we compared a commercial sequence, targeting mouse mRNA transcript (m)siCEBPB (Ambion, ID #s63860), with our own *in house* identified and synthesized human sequence (h)siCEBPB and with the mismatch converted sequence (h/m)siCEBPB (Table 1) (Figure 1(e)).

**Figure 2:**
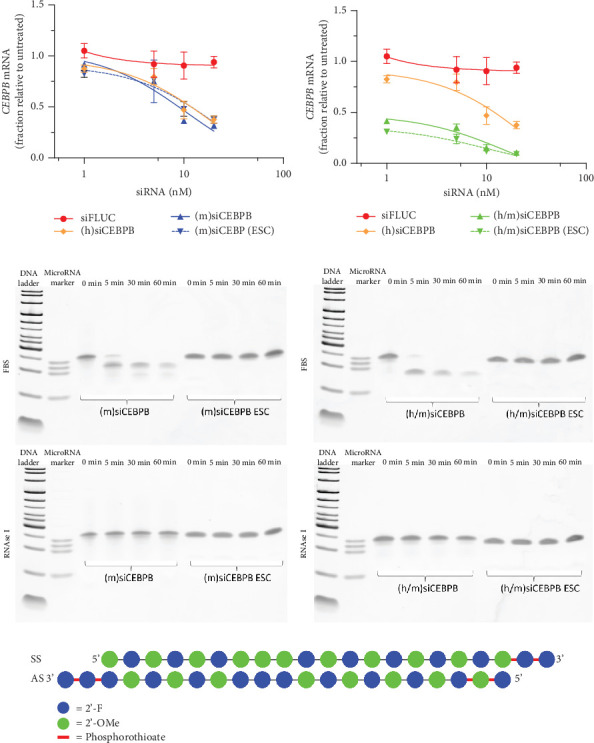
Sequence optimization and chemical stabilization of siCEBPB in BNL 1ME mouse cells. BNL 1ME cells were transfected 24 h after seeding, with 1, 5, 10, and 20 nM of human-specific (h)siCEBPB, mouse-specific (m)siCEBPB, or human–murine mismatch converted (h/m)siCEBPB sequences. Silencing effect of unmodified (naked) or enhanced stability chemistry (ESC) similar chemically modified (a) (m)siCEBPB and (b) (h/m)siCEBPB sequences, expressed as percentage CEBPB mRNA transcript knockdown, relative to siFLUC negative control. (c) Polyacrylamide gel–based early-stage stability assay of naked versus “ESC similar” chemically modified sequences, top panel for FBS and bottom panel for RNAse I-treated siRNAs. (d) Scheme of “ESC similar” chemical modification pattern, applied to sequences for improved stability. Statistics: *n* = 2, error bars represent mean ± SD.

Mouse BNL 1ME cells were transfected with 1, 5, 10, and 20 nM of siFLUC negative control or each one of the siCEBPB sequences human (h), mouse (m), and human/mouse (h/m) mismatch converted surrogate. Silencing activity of (h)siCEBPB and (m)siCEBPB followed a similar trend with ~70% knockdown of target CEBPB mRNA transcript at 20 nM after 48 h. The chemical modification pattern on (m)siCEBPB ESC did not seem to affect the potency of the sequence (Figure 2(a)). To test if siRNA potency would be affected by the mismatch conversion, cells were treated for dose response with (h/m)siCEBPB, which resulted in a clear increase in potency, with CEBPB mRNA knockdown effect above 60% at 1 nM and ~90% at 20 nM after 48 h, for both naked and (h/m)siCEBPB “ESC similar” modified sequence (Figure 2(b)).

To confirm that the “ESC similar” chemical modification pattern was effective in stabilizing (m)siCEBPB and (h/m)siCEBPB against nuclease degradation, sequences were incubated in the presence of FBS and RNAse I and tested for degradation on a polyacrylamide gel. RNAse I, which has a preference for single-stranded RNA, was employed to assess the extent of duplex formation and the stability of any unprotected single-stranded regions within the siRNA molecule (Figure 2(c)). Both sequences containing “ESC similar” pattern (Figure 2(d)) showed increased stability, as seen from almost absent short molecular weight degradation bands. Gain in metabolic stability was more pronounced for FBS than RNAse I treatment conditions, during the 1-h timeframe (Figure 2(c)).

### 3.3. TfR-siCEBPB Shows Potent Antitumor Effect in a Pancreatic Cancer Liver–Metastatic Mouse Model of PDAC

To test the antitumor effect of the siCEBPB aptamer conjugates in advanced PDAC, a traceable animal model was used. Two weeks after PANC-Luc2 xenograft implantation and prior to first injection, animals were weighted and luciferase expression was measured, as number of photons emitted per animal, using an IVIS optical imaging system. Average animal weight was 25.1 g and average photon count was 6.9 × 10^6^ photons per animal (data not shown).

**Figure 3:**
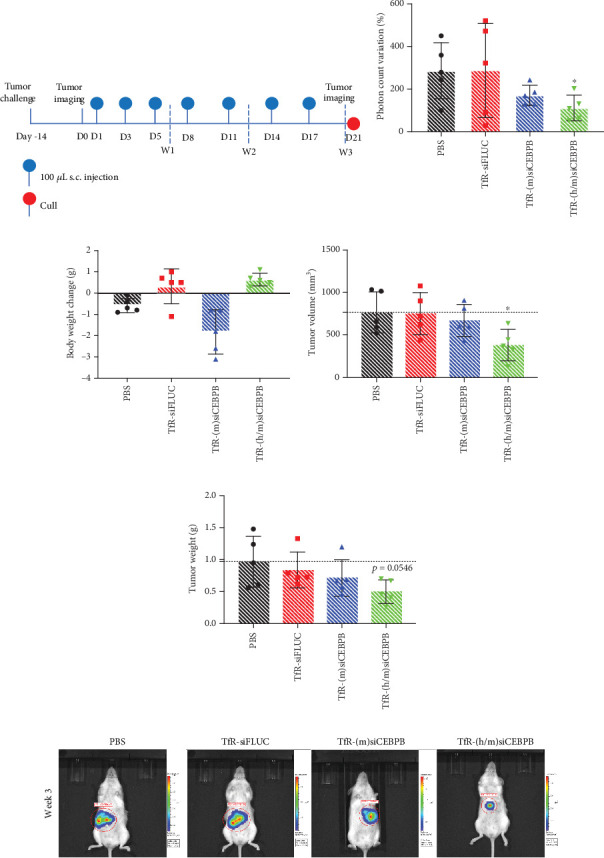
Effect of TfR-siCEBPB conjugates in a pancreatic cancer liver–metastatic mouse model. NOD/SCID mouse liver xenografted with luciferase-expressing human pancreatic cancer cells (PANC-1-Luc2) had tumors developing for 2 weeks and then injected s.c. with PBS, 15 mg/kg of TfR-siFLUC, TfR-(m)siCEBPB, or (h/m)TfR-siCEBPB, at Days 1, 3, 5, 8, 11, 14, and 17 and culled on Day 21. (a) Scheme of treatment protocol. (b) Tumor bioluminescence variation, as percentage of tumor emitted photons between Day 1 and Day 18. (c) Body weight variation between Day 1 and Day 21. Livers were excised and dissected out tumors were measured for calculation of (d) volume and determination of (e) weight. (f) Representative IVIS spectrum in vivo images, obtained after 3 weeks of treatment. Statistics: asterisks denote significance in respect to the PBS group, as follows: ⁣^∗^*p* < 0.05, ⁣^∗∗∗^*p* < 0.001; unpaired *t*-test with Welch's correction; mean ± SD.

On Day 1, animals were injected s.c. with 100 *μ*L of PBS, TfR-siFLUC, TfR-(m)siCEBPB, or TfR-(h/m)siCEBPB at a 15 mg/kg dose. Injections were repeated on Days 3, 5, 8, 11, 14, and 17 and animals culled on Day 21. Tumor growth was evaluated by measurement of bioluminescence intensity signal at beginning and just prior to culling (Figure 3(a)).

Animals from the group treated with TfR-(h/m)siCEBPB showed a significantly lower average variation of tumor emitted photons, when compared to control (112% vs. 286%) (Figures 3(b) and 3(f)). Despite showing a decrease in the bioluminescence read, the group treated TfR-(m)siCEBPB did not perform as well in terms of tumor reduction (Figures 3(d), 3(e), and 3(f)). Interestingly, for two of the animals from the siFLUC control group, the bioluminescence decreased drastically, which suggests loss of luciferase expression. However, due to tumor growth, this effect is difficult to evaluate (Figures 3(b) and 3(f)). Also a sign of a differential effect from the treatment is the observation that animals treated with TfR-(h/m)siCEBPB increased their body weight. That change is small when compared to the TfR-siFLUC negative control but highly significant (*p* = 0.0003) when compared to the saline-treated group (Figure 3(c)).

Tumor tissue, harvested after culling, was measured for its volume and weight. Consistently, TfR-(h/m)siCEBPB-treated animals showed, on average, significantly smaller tumor volume (~370 mm^3^) when compared to control (~760 mm^3^), which represents a reduction of ~50% of tumor size (Figure 3(d)). This result is accompanied by a smaller tumor weight observed for animals from same group, which show approximately half of the weight (~0.5 g) when compared to control (~1 g) (Figure 3(e)). In summary, data shows that TfR-(h/m)siCEBPB is the best performing therapeutic sequence in the PDAC mouse model.

### 3.4. TfR-CEBPB Treatment Results in Target mRNA Knockdown in Liver and Tumor of a Pancreatic Cancer Liver–Metastatic Mouse Model of PDAC

**Figure 4:**
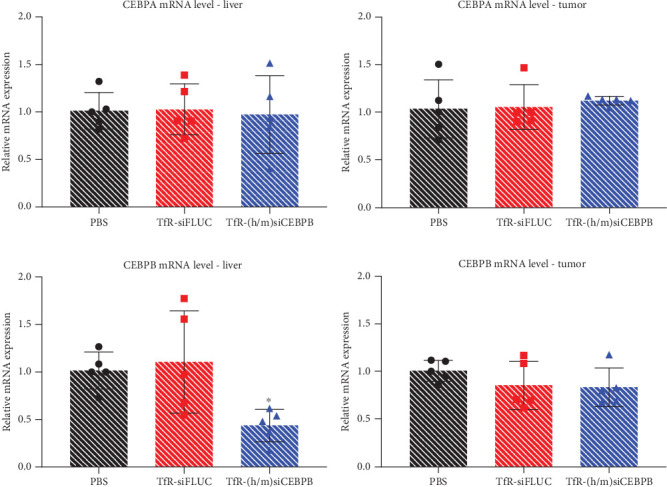
Effect of TfR-siCEBPB on CEBPA and CEBPB mRNA levels in liver and tumor tissue in a pancreatic cancer liver–metastatic mouse model of PDAC. Liver and tumor tissue samples, collected from five of the NOD/SCID PDAC model mice, from each group, treated with 15 mg/kg of (h/m)TfR-siCEBPB s.c., were analyzed to determine knockdown of CEBPA and CEBPB mRNA transcript. CEBPA mRNA expression relative to untreated control in (a) liver and (b) tumor. CEBPB mRNA expression in (c) liver and (d) tumor. Statistics: asterisks denote significance in respect to the PBS group, as follows: ⁣^∗^*p* < 0.05; unpaired *t*-test with Welch's correction; mean ± SD.

To confirm target engagement and pharmacodynamics for the TfR-siCEBPB conjugate on the PDAC animal model, liver and tumor tissues were processed and analyzed for CEBPA and CEBPB mRNA expression. PD analysis was limited to the group which showed statistically significant tumor reduction (Figures 3(a) and 3(d))—TfR-(h/m)siCEBPB, highlighting the biological relevance of CEBPB mRNA expression patterns and their implications in the therapeutic efficacy. Despite a subtle increase in expression in the tumor tissue and positive variation for some of the animals in liver, mRNA levels for CEBPA do not change significantly with the treatment, in any of the tissues (Figures 4(a) and 4(b)). Importantly, CEBPB mRNA knockdown was evident in liver tissue, with average transcript downregulation of ~45%, along with an apparent ~25% knockdown in tumor tissue, relative to the saline control group (Figures 4(c) and 4(d)).

### 3.5. In Vivo Delivery of TfR-CEBPB Reduces Tumor Growth in DEN-Induced Cirrhotic HCC

**Figure 5:**
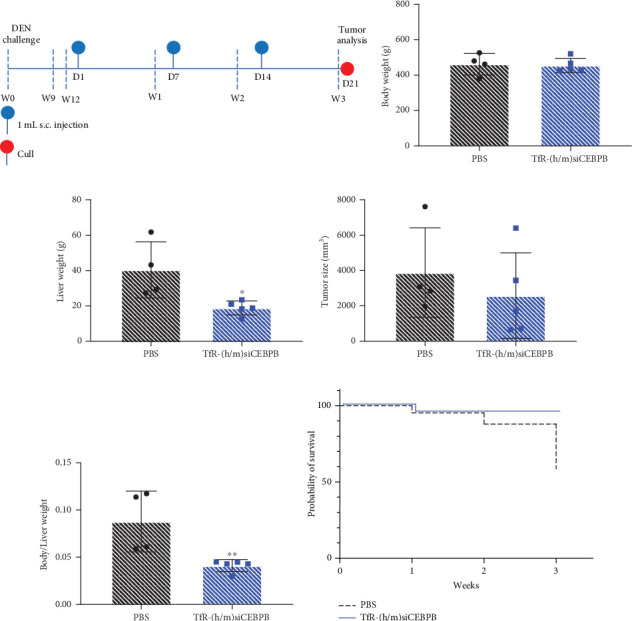
In vivo activity of TfR-siCEBPB in DEN-induced cirrhotic HCC rat model. DEN-exposed male Wistar rats, which developed spontaneous liver tumors after 12 weeks, were treated via s.c. injection with 15 mg/kg of (h/m)TfR-siCEBPB, once weekly, for 3 weeks. (a) Study protocol scheme. (b) Animal body weight, (c) liver weight, (d) tumor size, and (e) liver/body weight at the end of treatment. (f) Survival rate for untreated and treated group of eight animals, during the 3-week period. Statistics: asterisks denote significance as follows: ⁣^∗^*p* < 0.05, ⁣^∗∗^*p* < 0.01; unpaired *t*-test with Welch's correction; mean ± SD.

Treatment of DEN-exposed male Wistar rats with 15 mg/kg of (h/m)TfR-siCEBPB, once weekly, for 3 weeks (Figure 5(a)), did not result in measurable difference in total body weight (Figure 5(b)). Importantly, a lower liver weight of ~20 g was measured for the treated cohort (Figure 5(c)). Tumor size followed the same trend, with ~50% decrease for the treated animals, when compared to saline-treated control (Figure 5(d)). The ratio of liver to body weight of ~0.05 reveals a decrease in liver tissue growth and tumor burden for the (h/m)TfR-siCEBPB-treated animals (Figure 5(e)). Probability of survival was monitored during the 3-week treatment plan. From the eight animals in each group at the start of treatment, seven on the (h/m)TfR-siCEBPB-treated group survived for the entire duration of the study, in comparison to only four on the saline-injected control group, which translates into a probability of survival, for the 3-week period, of ~90% and ~50%, respectively (Figure 5(f)).

### 3.6. Activity of TfR-siCEBPB Improves Serum Biomarkers for Liver and Pancreatic Function

**Figure 6:**
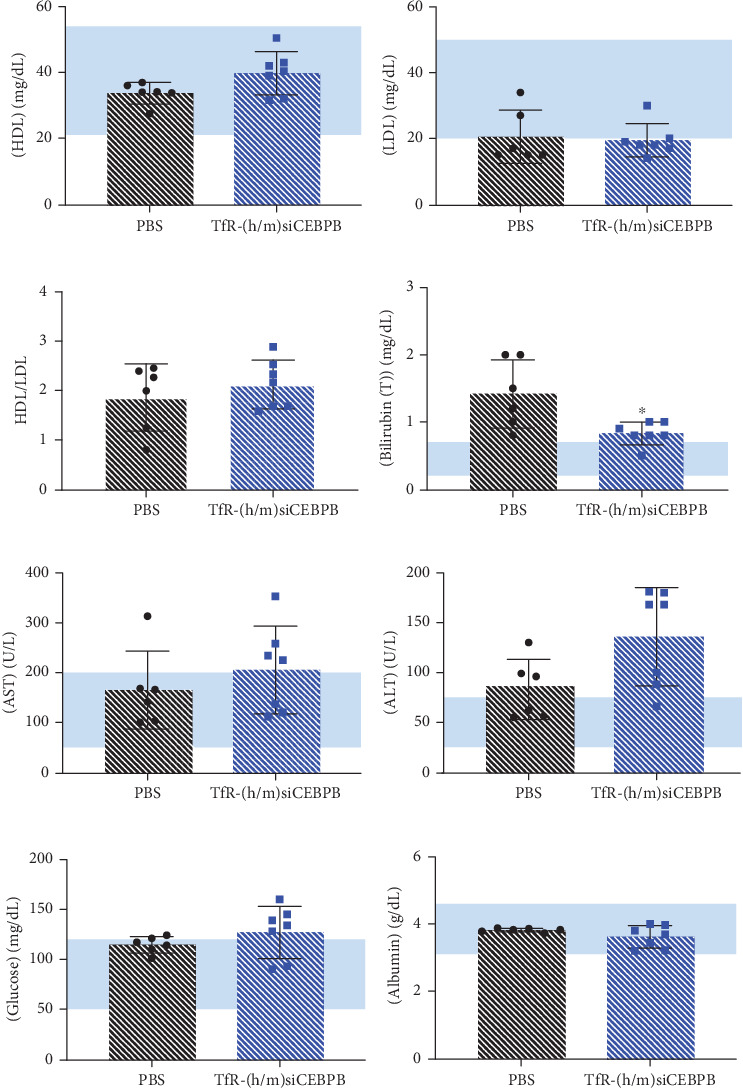
Serum biomarkers for liver and pancreatic function after treatment with TfR-siCEBPB. Serum from the DEN-exposed male Wistar rats was collected from all animals at time of culling and analyzed for (a) HDL cholesterol, (b) LDL cholesterol, and resultant (c) HDL/LDL ratio. Liver function assessed for (d) bilirubin, (e) alanine aminotransferase (AST), and (f) aspartate aminotransferase (ALT) levels. (g) Glucose and (h) albumin serum concentrations were also determined and compared between (h/m)TfR-siCEBPB-treated and untreated cohorts. Cyan rectangles delimit the normal range for each of the biomarkers [39]. ⁣^∗^*p* < 0.05; unpaired *t*-test with Welch's correction; mean ± SD.

The effect of (h/m)TfR-siCEBPB on liver function was assessed, with serum HDL and LDL cholesterol showing small variations of 10 mg/dL for HDL and almost no change on LDL, in comparison to the PBS control group, with an overall HDL/LDL ratio of ~2 (Figures 6(a), 6(b), and 6(c)). Decreased bilirubin levels, from 1.3 in untreated to 0.8 mg/dL in the treated group, suggest a tendency to normalization, considering the reference total bilirubin for Wistar rats 0.8–1.3 mg/dL (Figure 6(d)) [39]. Variation of AST (160 vs. 200 U/L) and ALT (60 vs. 125 U/L) levels is nonsignificant (Figures 6(e) and 6(f)) [39]. A small variation in serum GLU (120 vs. 125 mg/dL) (Figure 6(g)) and stable, within normal range ALB levels (3.8 vs. 3.7 g/dL) [39], were observed for the treated animals compared to the untreated (Figure 6(h)). These results suggest that, in the rat cirrhotic HCC model, the therapy was well tolerated, without causing a significant transaminitis or bilirubin increase.

## 4. Discussion

In this work, we show evidence that a nucleic acid–based therapy, formed by the combination of a TfR targeting RNA aptamer and an siRNA for CEBPB, has potential for treatment of both primary tumor and metastatic lesions in the liver. Aptamers have emerged as an interesting method to target cancer cells and have some advantages compared to antibodies, including low immunogenicity and toxicity, structural stability, can be chemically synthesized [40], and importantly allow subcutaneous administration route to be considered. The transmembrane protein TfR is reported to be overexpressed up to 100-fold in cancer cells [22] and is strongly correlated with a malignant phenotype, both in PDAC [24] and HCC [23, 41], making these excellent therapeutic targets for an aptamer conjugate.

In a previous study, focused on determining the antitumor effect of an aptamer-saRNA conjugate, targeting liver metastasized advanced PDAC, we provided evidence that delivery of CEBPA-saRNA by a human TfR aptamer leads to potent antitumor effects, in a mouse model of advanced PDAC [27, 29]. In the referred study, animals treated with 1 mg/kg of TfR-saCEBPA, 3 times per week, for 3 weeks, lead to an mRNA upregulation of ~1.5-fold of CEBPA and a ~2.5-fold of its downstream target p21. These effects were correlated to a significant reduction of ~1/3 of the tumor size, when compared to control [27, 29]. Based on these findings, we used the same chemical scaffold, that is, the same aptamer sequence and modification patterns, proven as optimal for functional and safe delivery of short, double-stranded therapeutic oligos, and took advantage of its modularity, by simply changing the therapeutic oligo component to a CEBPB siRNA.

The liver-enriched CEBPB gene can function as either an oncogene or a tumor suppressor gene depending on the cellular context. As an oncogene, CEBPB can promote tumor growth and progression by enhancing cell proliferation, energy metabolism, invasion, and growth and specifically in differentiation of myeloid and lymphoid cell survival [42–44]. On the other hand, in certain scenarios, CEBPB can act as a tumor suppressor gene by inducing cell differentiation, inhibiting cell proliferation, and promoting apoptosis [8]. The CEBPB gene can produce three distinct protein isoforms: LAP1, LAP2, and LIP [45, 46]. These isoforms have different impacts on the gene's role as an oncogene or tumor suppressor [18]. The LAP1 and LAP2 isoforms act as transcriptional activators, promoting the expression of genes involved in cell differentiation, growth arrest, and immune response [46]. Their tumor-suppressive effects are mediated through the regulation of cell cycle progression, apoptosis, and the inhibition of cell proliferation [18, 46]. On the other hand, the LIP isoform functions as a dominant-negative regulator on dimerization, inhibiting the activity of LAP isoforms and other C/EBP family members [46]. LIP promotes cell proliferation, survival, and invasion, and its overexpression has been associated with aggressive tumor behavior [46]. In summary, the fine translational controlled ratio of CEBPB expression determines cell fate, which in combination with their complex regulation, by dimerization with other C/EBPs isoforms and other bZIP family member proteins [47], may explain why CEBPA upregulation was not so pronounced in the PDAC mouse model analyzed tissues, where CEBPB downregulation was effectively measured.

As a downstream effector of Ras signaling, the transcription factor CEBPB represents an interesting therapeutic target in metastatic PDAC and HCC [23, 24, 41]. Analysis of human and rodent tumor cells has shown that CEBPB has pro-oncogenic functions and is essential for the development of many cancers [48]. It has been shown that colon adenocarcinoma cells displayed reduced tumorigenic potential when transplanted into CEBPB-deficient animals [48]. Furthermore, mice with oncogenic Ras tumors have been shown to be dependent on CEBPB for their survival with deletion of CEBPB in pre-existing oncogenic Ha-Ras mouse skin tumors in vivo, resulting in rapid regression [49], while, in an in vitro model of Ewing sarcoma, overexpression of CEBPB led to a significant increase in colony formation, which was depleted when CEBPB was deleted [50]. Furthermore, it was showed that overexpression also upregulated the cancer stem cell marker ALDH1A1, which confers resistance to chemotherapy [50].

siRNA therapy has previously been investigated in treatment of both PDAC and HCC. An siRNA targeting two different regions in the bcl-2 gene was shown to have antiproliferative and antiapoptotic effects, in a murine model, when injected intraperitoneally with siRNA seen to be quickly distributed to all organs and excreted via the kidney and liver [51]. The growth factor pancreatic adenocarcinoma upregulated factor (PAUF) is highly expressed in PDAC and has also been described as another target for RNA aptamer–based therapy [52]. Conjugation to chemically stabilized aptamers offers the advantages of preferential targeting tumor cells and thereby mitigating issues relating to half-life or off target effects of the siRNA.

We have specifically chosen to develop a TfR-siCEBPB aptamer conjugate for the treatment of metastatic PDAC and HCC. In previous studies, we have shown effective cellular uptake of Cy3-labeled TfR aptamer into various cancer cell lines [27, 29]. Moreover, we also reported positive therapeutic impact of TfR-saRNA conjugates in vivo, in different liver disease models [27, 28]. In this study, we designed and optimized a TfR-siCEBPB aptamer conjugate, which was evaluated in a primary liver cancer rat model of DEN-induced cirrhotic HCC and in a secondary liver cancer of metastatic PDAC, in which pancreatic cancer cells were directly implanted into the liver. Importantly, we identified a potent human/murine cross-reactive siRNA sequence—(h/m)siCEBPB, obtained using a strategy known as single mismatch conversion [53], using the (h)siRNA as template. The siRNA target region is highly conserved between human, mouse, and rat genomes, while the siRNA guide strand single mismatch is outside the seed region, which might explain the higher cross-species performance of (h/m)siCEBPB. We show that the aptamer conjugate has a robust silencing effect, in in vitro human and murine cells, reaching a potent reduction in CEBPB mRNA transcript of ~90%, when transfected at low nanomolar range. When tested by passive uptake, the conjugate still reached 50% knockdown at ~500 nM. Importantly, the effect was associated with a substantial oncological in vivo response, resulting in a robust tumor burden reduction, in both models, in all treated animals. After treatment with TfR-siCEBPB, downregulation of CEBPB mRNA in the liver and tumor tissue of metastatic PDAC model animals was evident. The tendency of serum biomarkers for the HCC model animals treated with the conjugate suggests that the therapy was well tolerated in cirrhotic animals, without causing transaminitis or features of liver decompensation.

## 5. Conclusion

We have developed a novel aptamer–siRNA-based therapy, targeting liver metastatic PDAC and HCC, which leverages overexpression of TfR for cancer cell delivery and the critical role of the transcription factor C/EBP*β* in tumor development. We have shown on target delivery in vitro and a significant therapeutic effect in two relevant in vivo murine disease models a liver metastatic mouse model of PDAC and a DEN-induced cirrhotic HCC rat model. The TfR-siCEBPB aptamer is a promising therapeutic option to take forward into clinical development, for treatment of both primary tumor and metastatic lesions in the liver.

## Figures and Tables

**Table 1:** Sequences of siRNAs and TfR aptamer.

**Sequence ID**	**Sense (5**⁣′**➔3**⁣′**)**	**Antisense (5**⁣′**➔3**⁣′**)**
siFLUC	CUU ACG CUG AGU ACU UCG AUU	UCG AAG UAC UCA GCG UAA GUU
(m)siCEBPB	GCA CCC UGC GGA ACU UGU UTT	AAC AAG UUC CGC AGG GUG CTG
(h)siCEBPB	CUG AGU AAU C**G**C UUA AAG AUU	UCU UUA AG**C** GAU UAC UCA GUU
(h/m)siCEBPB	CUG AGU AAU C**A**C UUA AAG AUU	UCU UUA AG**U** GAU UAC UCA GUU
TfR aptamer	UUU AUU CAC AUU UUU GAA UUG A(C3)(C3) (C3)(C3)(C3)	
*Internal (C3) spacer modification*		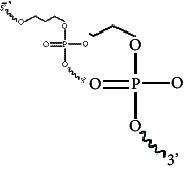

## Data Availability

The data that support the findings of this study are available from the corresponding author upon reasonable request.
