# Corrigendum to “Design and Evaluation of RNA Aptamer–Mediated Delivery of C/EBP*β* siRNA for Oncological Therapy”

**DOI:** 10.1155/jna/9867151

**Published:** 2025-09-10

D. Vasconcelos, M.H. Sodergren, V. Reebye, et al., “Design and Evaluation of RNA Aptamer–Mediated Delivery of C/EBP*β* siRNA for Oncological Therapy,” *Journal of Nucleic Acids* 2025 (2025): 1461574, https://doi.org/10.1155/jna/1461574

In the article, there is an error in the *y*-axis label for Figure 5e, which should read “liver/body weight.” The correct Figure 5 is shown below.

Additionally, the title of Section 2.5, “Animal Models, Experimental Design, and Sample Collection,” should read “Animal Care.”

We apologize for these errors.

## Figures and Tables

**Figure 1:**
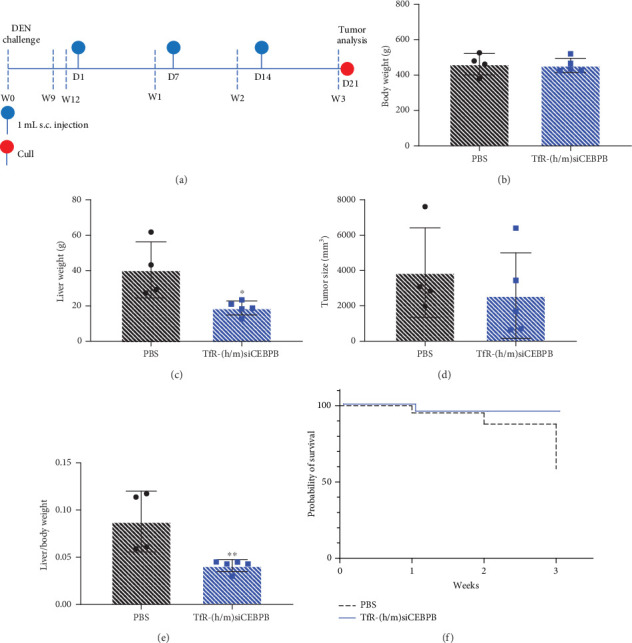
In vivo activity of TfR-siCEBPB in DEN-induced cirrhotic HCC rat model. DEN-exposed male Wistar rats, which developed spontaneous liver tumors after 12 weeks, were treated via s.c. injection with 15 mg/kg of (h/m)TfR-siCEBPB, once weekly, for 3 weeks. (a) Study protocol scheme. (b) Animal body weight, (c) liver weight, (d) tumor size, and (e) liver/body weight at the end of treatment. (f) Survival rate for untreated and treated group of eight animals, during the 3-week period. Statistics: asterisks denote significance as follows: ⁣^∗^*p* < 0.05, ⁣^∗∗^*p* < 0.01; unpaired t-test with Welch's correction; mean ± SD.

